# Simulation of Imatinib Pharmacokinetics in pregnant women with chronic myeloid leukemia in the third trimester

**DOI:** 10.1007/s00228-026-04049-z

**Published:** 2026-04-11

**Authors:** Paola Mian, Christianne A. R. Lok, Anouk E. W. K. Dontje, Jelmer R. Prins, Thijs H. Oude Munnink, Laurens Nieuwenhuizen, Sanne J. Gordijn, Daan J. Touw, Paul Malik

**Affiliations:** 1 Department of Clinical Pharmacy and Pharmacology, University Medical Center Groningen and University of Groningen, Hanzeplein 1, Groningen, 9713 GZ The Netherlands; 2 Department of Pediatrics, Beatrix Children’s Hospital, University of Groningen, University Medical Center Groningen, Groningen, Netherlands; 3 Pharmacometrics Expertise Center Of the Northern Netherlands, University Medical Center Groningen, University of Groningen, Groningen, The Netherlands; 4 Department of Pharmaceutical Technology and Biopharmacy, University of Groningen, Groningen, the Netherlands; 5 Department of Gynaecological Oncology, Centre of Gynaecological Oncology Amsterdam, Location Antoni van Leeuwenhoek - Netherlands Cancer Institute, Amsterdam, the Netherlands; 6 Department of Obstetrics and Gynecology, University of Groningen, University Medical Center Groningen, Groningen, Netherlands; 7 Department of Internal Medicine Máxima MC Veldhoven/Eindhoven, Veldhoven/Eindhoven, The Netherlands; 8 Department of Pharmaceutical Analysis, Groningen Research Institute of Pharmacy, University of Groningen, Groningen, The Netherlands; 9 Ionis, Carlsbad, USA

**Keywords:** Pregnancy, Imatinib, Pharmacokinetics

## Abstract

Purpose

To simulate different dosing regimens of imatinib in third trimester pregnant women with CML that could meet plasma exposure targets for efficacy (C_min_≥1000 ng/mL) without compromising on fetal safety risk.

Methods

An initial physiologically-based pharmacokinetic (PBPK) model from Loer et al. was verified with routine non-pregnant PK data from a single center and then scaled to pregnancy by implementing pregnancy physiological and enzymatic changes relevant to imatinib PK. The pregnancy model was evaluated by comparing predictions with PK data observed in pregnant women receiving imatinib 400 mg once daily (QD). Simulations explored different dosing regimens that would meet C_min_≥1000 ng/mL without meaningfully higher AUC_0-24h_.

Results

Predictions for imatinib PK in the third trimester were well aligned with the observed data. Simulations indicate that 14.4% of third trimester pregnant women would achieve plasma C_min_≥1000 ng/mL at 400 mg QD, compared to 51.7% of non-pregnant female comparators receiving the same dose.

Conclusions

While dividing 400 mg QD into 200 mg twice daily (BID) could modestly improve PK target attainment for third trimester pregnant women (28.5%), a dose of 300 mg BID could be needed to match C_min_ target attainment (54.2%) and AUC_0-24h_with expectations for a non-pregnant population receiving 400 mg QD. However, given the potential for increased fetal exposure, this approach requires careful risk-benefit assessment, and further research is warranted to establish the safest and most effective strategy.

## Introduction

As targeted therapies, tyrosine kinase inhibitors are often associated with improved efficacy and fewer serious adverse events when compared to traditional broad spectrum chemotherapy [1, 2]. Their improved safety profile, efficacy and convenient oral administration, resulted in increasing clinical experience with the use of these therapeutic agents in specific and vulnerable populations, such as pregnant women [3]. However, no tyrosine kinase inhibitor has been approved for cancer treatment in pregnant women. Few guidelines exist specified for pregnancy on the dosing and monitoring of safety outcomes for both the mother and the fetus. Pregnant women deserve access to optimal cancer therapy (at an optimal dose) that is supported by scientific evidence and agreed upon by clinicians and health authorities regarding the best benefit-risk ratio.

Imatinib is a tyrosine kinase inhibitor for which the body of literature is growing, along with relatively well-validated plasma target concentrations for effective and safe dosing or therapeutic drug monitoring (TDM) [4]. Pregnancy and cancer related outcomes after imatinib treatment in over 200 mothers with chronic myeloid leukemia (CML) have been documented, mostly at a dose of 400 mg once daily (QD) [3, 5–8]. Imatinib crosses the placental barrier in detectable amounts, and major malformations occur when imatinib is administered during organogenesis (i.e., in the first trimester). A consensus agreement indicates that imatinib can be used when benefits outweigh risks to the mother and fetus in the second and third trimesters, and should be used with caution [3, 6].

Many drugs require dose adjustment in pregnancy due to altered pharmacokinetics (PK) and/or pharmacodynamics (PD) [9]. Pregnancy is associated with significant anatomical and physiological changes which affect the PK of drugs and alter dosing requirements, such as increased blood volume and kidney perfusion, increased body weight, and changes in the expression/activity of metabolic enzymes in the liver [10–14]. In addition, precision medicine approaches such as TDM are increasingly preferred to achieve an efficacious dose for each pregnant patient that minimizes the fetal exposures as much as possible [4, 15, 16].

There is an unmet need to generate meaningful dosing guidance for imatinib pregnant women. Ultimately through better dosing guidance, fewer pregnant women will be exposed to potentially ineffective or unsafe doses (for either the mother or the fetus). Physiologically based pharmacokinetic (PBPK) models may be useful for dosing guidance in this setting since PK data from prospective clinical trials are difficult if not impossible to obtain [17, 18]. PBPK models combine knowledge of anatomy and physiology and drug-specific parameters (e.g., the physicochemical information) to parameterize equations for simulating the absorption, distribution, metabolism and elimination (ADME) of a compound in the body [19]. Pregnancy-PBPK models, which adapt the virtual body to account for the physiological effects of pregnancy (and which often add a mechanistic fetal compartment), are emerging to predict PK in this population [20–22]. When mechanistic predictions of PK in different stages of pregnancy are shown to be accurate following an evaluation against the available data, the pregnancy-PBPK models can be used with relative confidence to simulate dosing regimens that could optimize the maternal plasma exposures, and that could eventually support extrapolation of efficacy and safety (accounting for the fetal exposures, too) [17, 23].

This study aims to verify a PBPK model of imatinib against real-world non-pregnant PK comparator data from a single center, scale the model to pregnancy to simulate imatinib plasma PK in the third trimester, and then to simulate different dosing regimens in populations of third trimester pregnant women that could meet plasma exposure targets for efficacy (C_min_ ≥1000 ng/mL) without compromising on safety risk. Focus was on third trimester pregnant women only, as data were not available to inform PBPK simulations in the second trimester [24].

## Methods

### Clinical pharmacology and pharmacokinetics of imatinib

Imatinib is a tyrosine kinase inhibitor. It targets the Bcr-Abl tyrosine kinase. Inhibition of this enzyme blocks proliferation and induces apoptosis in Bcr-Abl positive cell lines as well as in fresh leukemic cells in Philadelphia chromosome positive CML. Imatinib also inhibits tyrosine kinase for platelet-derived growth factor (PDGF), stem cell factor (SCF), c-kit, and cellular events mediated by PDGF and SCF [25]. Imatinib is approved for the treatment of several types of leukemia such as Philadelphia chromosome positive CML, Philadelphia chromosome positive ALL, and chronic eosinophilic leukemia. It is further used to manage gastrointestinal stromal tumor (GIST), dermatofibrosarcoma protuberans, myelodysplastic/myeloproliferative neoplasms and systemic mastocytosis. Following oral administration, imatinib is well absorbed from the gastrointestinal tract with an oral bioavailability of 98%.^26^ Food does not have a meaningful effect on the rate or extent of its bioavailability [26]. Imatinib is 95% bound to albumin and alpha_1_-acid glycoprotein and distributes adequately throughout the body tissues, with an estimated volume of distribution of approximately 400 L.^27^ The median terminal plasma half-life in adults with cancer is approximately 18 h. [27] Metabolism is predominantly carried out by CYP3A4 and CYP2C8 [28, 29]. Total drug material is excreted mostly in feces, with a five-times higher amount of the dose in feces than in urine [30]. P-glycoprotein (P-gp) is involved in the excretion of imatinib unchanged to the bile and urine [28, 29]. Due to mechanism-based autoinhibition of CYP3A4, the contribution of CYP3A4 to imatinib metabolism declines from the first dose to steady state [28].

### Retrospective chart review to collect routine pharmacokinetic data

Routine steady state imatinib plasma concentrations along with sampling time since the last dose administration were obtained from the electronic patient system from one academic hospital (University Medical Center Groningen [UMCG]). TDM of imatinib is standard of care within the Netherlands, to optimize therapy [31]. Informed consent was waived by the UMCG medical ethics committee (METc 2024/152). Plasma concentrations, sample conditions and basic demographic variables were collected by retrospective chart review.

### PBPK modeling software

PBPK modeling and simulation were performed using the Open Systems Pharmacology Suite, version 11.2 (PK-Sim^®^ and Mobi^®^, version 11.2, https://www.open-systems-pharmacology.org/). The pregnancy-PBPK model was built in the companion tool, MoBi^®^ and exported back to PK-Sim^®^ for population simulations. WebPlotDigitizer (https://automeris.io/WebPlotDigitizer/) was used to extract PK data from literature. R (version 4.3) was used for data visualization and statistical analysis.

### Verification of the non-pregnant PBPK model

A comprehensive PBPK model for imatinib and its major metabolite (desmethyl-imatinib) was developed by Loer et al. for non-pregnant adults with cancer, capturing the major victim and perpetrator drug-drug interactions [28]. This model was used with no modifications for the current work. The original model was calibrated using PK data from healthy subjects and patients with cancer following single and multiple doses from literature. Verification against the drug-drug interaction studies ensures that the model has the correct sensitivity to changes in expression/activity of CYP3A4 and CYP2C8. Imatinib itself is a mechanism-based inactivator of CYP3A4, and this process was accounted for in the simulations [28].

For this work, a (re)-verification of the imatinib model in non-pregnant adults was performed by predicting the multiple dose PK of imatinib in a population of cancer patients at the UMCG center, which underwent TDM of imatinib as part of clinical care. PK predictions were performed using a simulated population that matched the anthropometric measures of the UMCG population (by age, body weight, and European ancestry) (Table 1). Cancer patients were otherwise not set to be different than healthy adults of the same demographic characteristics, as the presence of cancer does not appear to impact the PK of imatinib (as deduced by Loer et al. and others) [28, 29].

**Table 1:** Pharmacokinetic datasets for imatinib model verification and evaluation in pregnancy

Study	Dose and Administration	Cohort	*N*	Age (years)	Weight (kg)	Remarks
Non-Pregnant Model Verification						
TDM data from UMCG	oral capsule multiple dose 400 mg/day	NL: CML and GIST	42	67.32 ± 11.85 ^**a**^	78.72 ± 19.79 ^**a**^	**-**
**Pregnant Model Evaluation**						
Ali et al., 2009^35^	oral capsule multiple dose 400 mg/day	TR: Philadelphia chromosome positive CML	1	27	NR	Delivery at 39th week GA
Burwick et al., 2017^34^	oral capsule multiple dose 400 mg/day	US: chronic-phase CML	1	29	NR	Delivery at ~ 38th week GA
Russell et al., 2007^36^	oral capsule multiple dose 400 mg/day	US: Philadelphia chromosome positive CML	1	NR	NR	Delivery at term

^a^Mean ± SD reported. Abbreviations: *CML* chronic myeloid leukemia, *GIST* gastrointestinal stromal tumor, *GA* gestational age, *TR* Turkey, *US* United States of America

### Development of the pregnancy PBPK model

The adult model for oral intake of imatinib was scaled to pregnant women to predict steady state imatinib exposure in the third trimester following 400 mg QD and other dosing regimens. All drug-specific inputs, were extrapolated to pregnant women by substituting the standard model structure with the pregnancy model structure, which includes nine additional compartments [22]. The demographic measures of the virtual pregnant women matched those of the in vivo study group, if the latter were reported. If not reported, the mean demographic measures available in PK-Sim^®^ were used.

### Enzymatic and transporter changes during pregnancy

Primary literature was screened for quantitative information on pregnancy-related changes in the expression and activity of the major enzymes and transporters that affect imatinib disposition (namely CYP3A4, CYP2C8, and P-gp for this work). CYP3A4 expression/activity is induced to 160% in the third trimester compared to non-pregnant women according to the aggregate modeling from Dallmann et al [32]. Only minimal information and no consensus is available in the literature regarding in vivo changes of CYP2C8 expression/activity in a gestational age (GA)-dependent manner, it was assumed that no inhibition or induction of CYP2C8 by pregnancy state occurred. Induction of P-gp expression/activity to 187% at 28 to 32 weeks GA was reported by Hebert et al. as derived from the percentage increase in unbound clearance of digoxin, a sensitive substrate of P-gp, as compared to when measured at 6 to 10 weeks post-partum [33]. This value was extrapolated linearly from 30 weeks GA to the later phases of the third trimester (mean + 99% at 34 weeks GA and mean + 110% at 38 weeks GA).

### Evaluation of the pregnancy PBPK model

Evaluation of the pregnancy PBPK model was conducted for the third trimester only, as no observed data for the second trimester could be identified in the literature. The scaled model was evaluated using observed imatinib plasma concentration datapoints collected from three studies (Table 1). These studies measured imatinib PK in three women at term (~ 38–39 weeks of GA) following oral dosing of 400 mg QD [34–36]. For comparison, the aggregated geometric mean PK profile and 5th to 95th prediction interval for a population of 1000 pregnant women in the latter part of the third trimester (ranging from 35 to 41 weeks GA) was simulated.

### Dosing simulations throughout pregnancy

Dosing simulations were run at 400 mg QD, 200 mg twice daily (BID, every 12 h), and 300 mg BID for populations of 1000 virtual pregnant women in the third trimester (29 to 31 weeks GA) and 1000 virtual age-matched non-pregnant female comparators. Imatinib 400 mg QD is the dose almost exclusively reported as being used during pregnancy [6, 7]. Simulations for the first trimester of pregnancy were not performed since imatinib is contraindicated due to risk of fetal malformations. Simulations for the second trimester of pregnancy were not performed since no information was available to inform the model in that stage (no literature data available for evaluation). Maximum concentration (C_max_), area under the plasma concentration time curve from time zero to 24 h after the morning dose (AUC_0−24 h_), and minimum concentration over the 24-hour interval (C_min_) were reported after simulation to steady state. Summary statistics were geometric mean, arithmetic mean, and arithmetic coefficient of variation.

There is growing evidence that sustained imatinib plasma concentrations ≥ 1000 ng/mL is a suitable exposure target for optimizing the benefit-risk ratio for non-pregnant patients with CML when using TDM or precision dosing methods [4, 37–39]. For each simulation, the percentage of the virtual population achieving the C_min_ target concentration was reported.

### Sensitivity analysis

To account for potential uncertainty in the assigned effects of pregnancy on CYP3A4 and P-gp expression/activity, a matrix block sensitivity analysis was performed testing the null hypothesis, the current hypothesis, and double the current hypothesis on each enzyme/transporter. Pregnancy population simulations were developed with each combination of changes for CYP3A4 and P-gp (8 permutations in total, with the ninth being the current model). Target attainment was reported for each assumption following imatinib 400 mg QD.

## Results

### Verification of the non-pregnant PBPK model

**Fig. 1:**
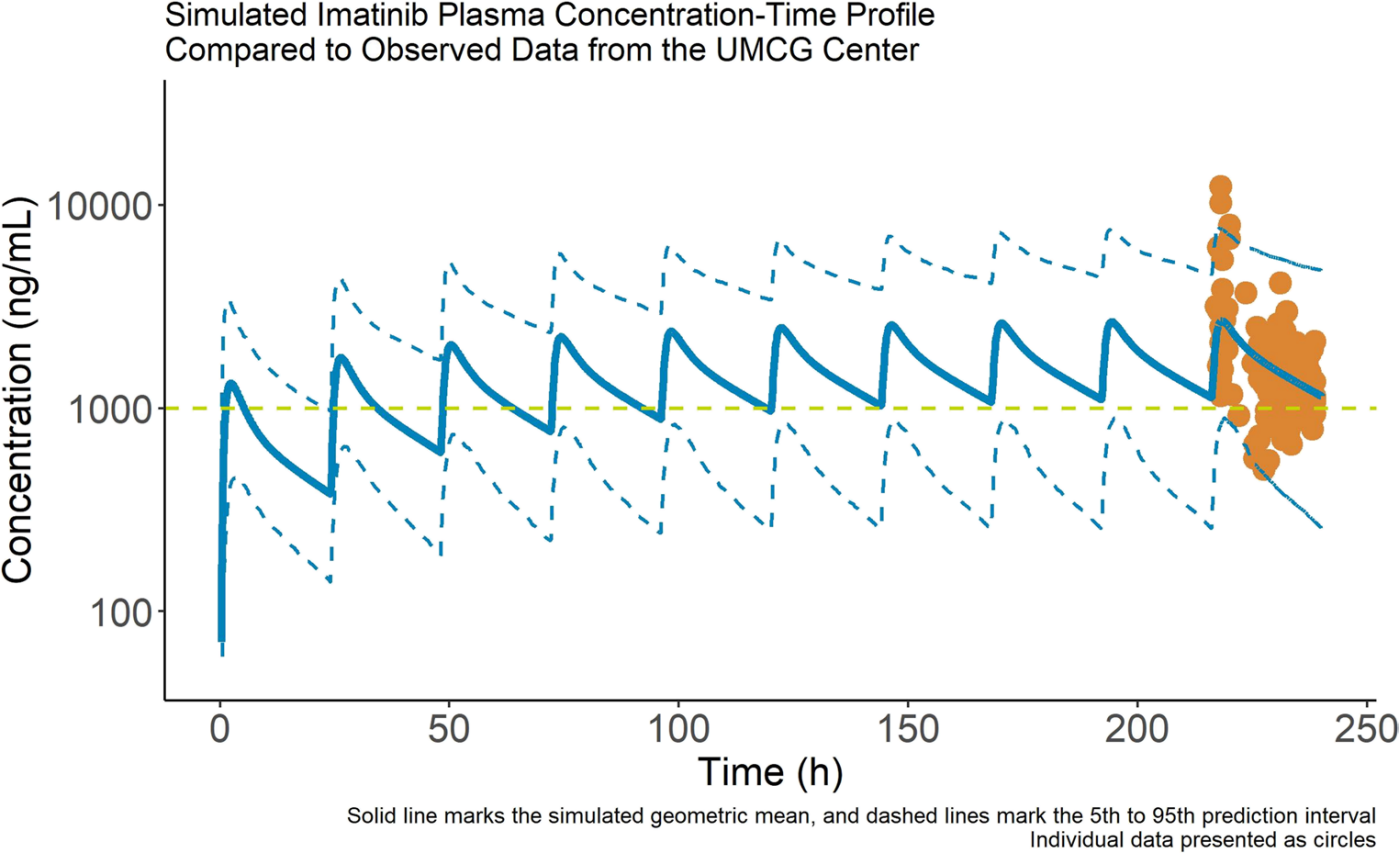
Verification of the imatinib PBPK model for predicting PK in non-pregnant cancer patients (CML, GIST)

A comparison between the observed PK measured in the ‘real world’ TDM data and the estimated steady state PK profile for cancer patients is presented in Fig. 1. The geometric mean fold error, comparing the geometric mean prediction to the individual imatinib concentrations, was 0.92. The average fold error for the same comparison was 1.03, suggesting that the individual concentrations are well-distributed above and below the prediction line at all time points. All but three of the individual observed datapoints near C_max_ fell within the 90% prediction interval for the population.

### Evaluation of the pregnancy PBPK model

**Fig. 2:**
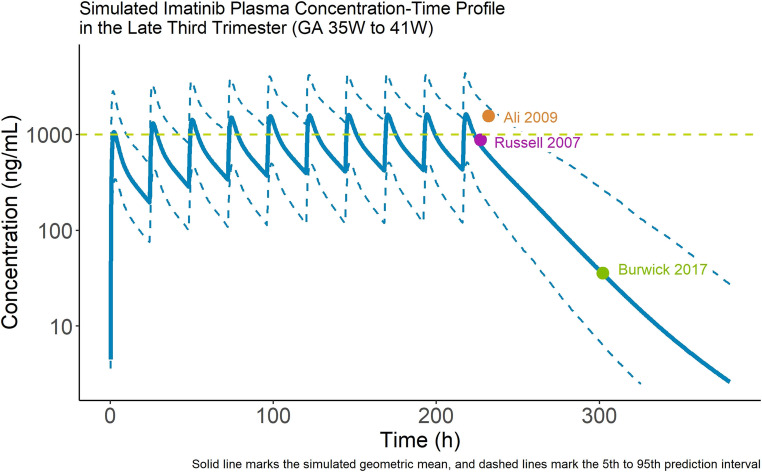
Evaluation of the imatinib Pregnancy-PBPK model for predicting PK in pregnant women with CML in the third trimester

A comparison between the observed PK from each study and the estimated PK profile in the latter phase of the third trimester nearing term is presented in Fig. 2. The simulations were well-aligned with the observed datapoints, but with a trend towards slight under-prediction of the plasma concentrations. All observed datapoints fell within the 90% prediction interval for the population. Two of the three individual points were predicted with better accuracy than the simulated geometric mean for the population (within 0.8- to 1.25-fold error). The geometric mean predicted vs. individual observed concentrations were 776 vs. 886 ng/mL at 11 h after dosing (0.88-fold error), 610 vs. 1562 ng/mL at 16 h after dosing (0.39-fold error), and 35.4 vs. 35.7 ng/mL at 86 h after dosing (0.99-fold error). Following this evaluation, the model was considered suitable to simulate the plasma PK of imatinib in populations of pregnant women in the third trimester.

### Dosing simulations throughout pregnancy

**Fig. 3:**
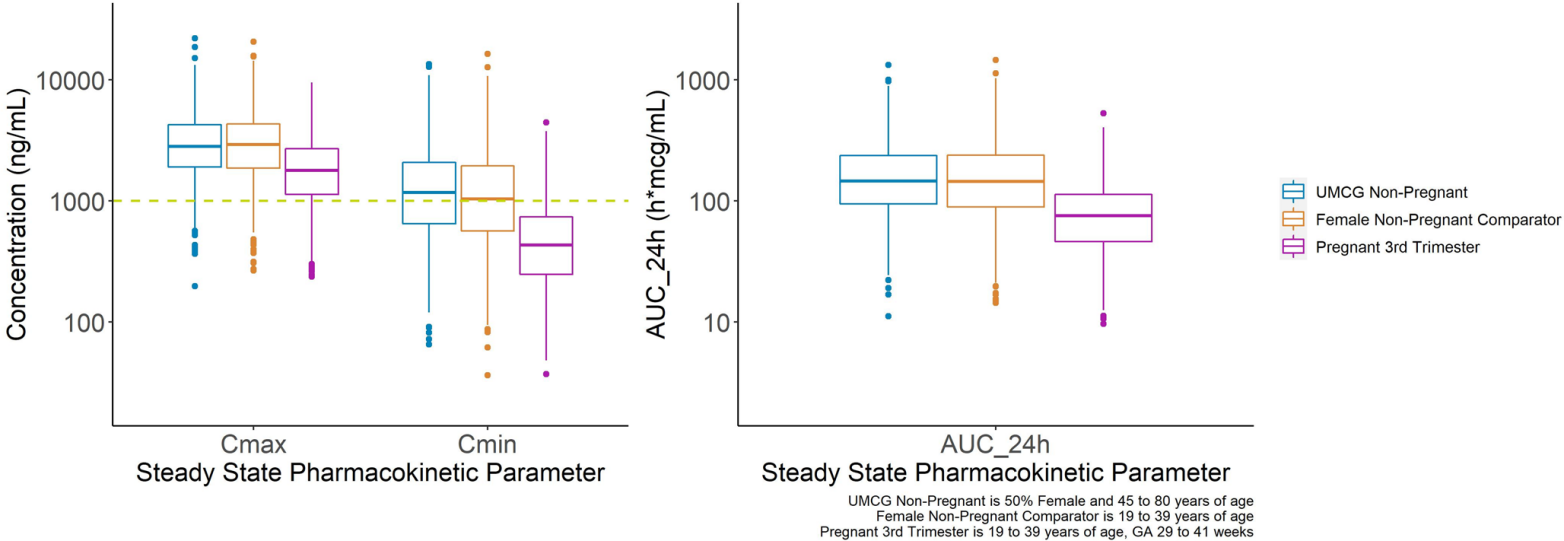
Simulated imatinib exposures following multiple oral dosing of 400 mg QD vs. efficacy and safety targets in non-pregnant cancer patients, pregnant women with cancer in the third trimester, and an age-matched female comparator group with cancer

The simulated plasma PK parameters and exposure target attainment in non-pregnant patients, the female age-matched non-pregnant comparators and third trimester pregnant women are shown in Table 2; Fig. 3. Following multiple oral dosing of 400 mg QD, the exposures in pregnant women in the third trimester are predicted to be largely subtherapeutic (85.6% of patients not achieving the target C_min_ ≥1000 ng/mL). In comparison, approximately 56.7% and 51.7% of UMCG non-pregnant and female non-pregnant comparators are predicted to achieve the target exposure, respectively at the same dose. These predictions for target attainment in the non-pregnant cancer population are representative of the target attainment rates from other recent real-world studies, as well as simulation studies [38–40].

**Table 2:** Simulated Geometric Mean (Mean, % Coefficient of Variation) Pharmacokinetic Parameters of Imatinib

Population	C_max, SS_	AUC_0−24,SS_	C_min, SS_	Percent C_min, SS_ ≥ 1000 ng/mL
	(ng/mL)	(h∙mcg/mL)	(ng/mL)	(%)
UMCG Non-Pregnant 400 mg QD	2793 (3417, 68%)	150 (192, 79%)	1148 (1666, 96%)	56.2%
Female Non-Pregnant Comparator 400 mg QD	2810 (3449, 66%)	144 (187, 79%)	1026 (1525, 100%)	51.7%
Pregnant 3rd Trimester 400 mg QD	1697 (2068, 62%)	72 (89, 68%)	425 (575, 85%)	14.4%
Pregnant 3rd Trimester 200 mg BID	1458 (1705, 57%)	87 (104, 63%)	673 (851, 74%)	28.5%
Pregnant 3rd Trimester 300 mg BID	2129 (2591, 63%)	131 (163, 69%)	1061 (1393, 78%)	54.2%

Modified dosing regimens were explored with additional simulations (Table 2). Dividing the 400 mg total daily dose into 200 mg BID may result in modest improvement in target attainment (28.5% vs. 14.4%) for pregnant women in the third trimester. However, simulations revealed that a further increase to 300 mg BID is likely required to match target attainment measures in pregnancy (third trimester) with target attainment observed in otherwise non-pregnant cancer patients (Table 2). Notably, the simulated AUC_0−24 h_ following 300 mg BID in pregnant patients meets and does not exceed the simulated AUC_0−24 h_ in the non-pregnant patients receiving 400 mg QD (i.e., suggesting no meaningful increase in safety/tolerability risk, but not yet considering maternal-fetal safety parameters).

### Sensitivity analysis

Results of the sensitivity analysis population simulations for target attainment for third trimester pregnant women are provided in Table 3. Changes up to + 198% for P-gp and changes up to + 120% for CYP3A4 (each being double the current assumptions) had approximately equivalent effects on imatinib trough concentration following 400 mg QD. In the null hypothesis scenario (no effects of pregnancy on either process), target attainment was still poor-to-modest in the third trimester population following 400 mg QD (42.5%).

**Table 3:** Sensitivity Analysis Accounting for Uncertainty Regarding Effects of Pregnancy on CYP3A4 and P-gp

Impact of pregnancy on *P*-gp expression/activity in the third trimester	Impact of pregnancy on CYP3A4 expression/activity in the third trimester		
	No Change	+ 60% (160% of reference)	+ 120% (220% of reference)
No change	42.5%	26.0%	14.1%
+ 99% (199% of reference)	26.0%	14.4%	7.0%
+ 198% (298% of reference)	15.7%	7.5%	3.3%

Table shows the simulated target attainment for a pregnancy population using each combination of assumptions for changes in CYP3A4 and P-gp. CYP3A4 = cytochrome P450 isozyme 3A4; P-gp = P-glycoprotein

## Discussion

In this study, a PBPK model was successfully developed to predict the PK of imatinib in third trimester pregnant women. This model was used to simulate dosing regimens (400 mg QD, 200 and 300 mg BID) that may optimize treatment of CML during third trimester of pregnancy. Models and simulations for first trimester were not developed, due to a contraindication to use this drug in this specific trimester. Models were also not developed for second trimester pregnant women, as there was no data available in literature to inform such simulations [24]. Simulations revealed that while dividing 400 mg QD into 200 mg BID could modestly improve PK target attainment for third trimester pregnant women (28.5%), a dose of 300 mg BID could be needed to match C_min_ target attainment (54.2%) and AUC_0−24 h_ with expectations for a non-pregnant population receiving 400 mg QD (51.7%).

There is a growing body of evidence for the maternal-fetal safety of imatinib 400 mg QD in the second and third trimesters. During organogenesis, studies in animals and in humans indicate that exposure of the embryo-fetus to imatinib can be harmful and must be avoided [41]. From the second and third trimesters, aggregate data suggest that imatinib may be used when benefits outweigh risks to the mother and fetus; imatinib is, one of the most studied drugs in pregnancy [6, 41]. In a review by Abruzzese et al., 265 reports of pregnant women were identified from literature of which 48% had normal birth, 21% unknown outcomes, 16% elective abortions, 9% spontaneous abortions, and 6% fetal abnormalities. [8, 24]. The latter two mainly occurred during the first trimester and during organogenesis. An international registry study of reports to the European LeukemiaNet describes similar results in pregnant patients. Twenty-one patients initiated imatinib therapy with successful childbirth – 13 patients in the second trimester and 8 patients in the third trimester. The median time of therapy initiation was 18 weeks GA, and the dose was mostly recorded at 400 mg QD. Complete hematological response information was available in 20 of those patients and was achieved in 19 (95%) at delivery. The single patient who did not achieve complete response only received imatinib from 32 to 35 weeks GA. Imatinib was well-tolerated by the women. One neonatal adverse event of intrauterine infection was noted for a mother-infant pair receiving imatinib and was not assessed as related to imatinib or the underlying disease (CML). Another child born prematurely at 35 weeks GA had a patent foramen ovale; her mother had received imatinib from week 33. Three infants exposed to imatinib had low birth weight (< 2500 g), but all recovered normal weight in the follow-up period and none needed medical intervention.

Maternal-fetal safety information is more limited at total daily doses above 400 mg. Intrauterine death at 7 months was reported for one mother-infant pair exposed to 600 mg QD and premature delivery with subsequent neonatal death was reported for two mother-infant pairs exposed to 800 mg QD.^3,7^ Accordingly the following treatment algorithm may be proposed: In female pregnancy patients for whom the benefits of treatment are considered to outweigh the risks, initiate therapy with imatinib 200 mg BID in the second trimester and if an indication toward complete hematological response is not observed by the third trimester, consider an increase to 300 mg BID. There is minimal indication for additional TDM of the plasma concentrations in this scenario because (1) response can be monitored directly from the Bcr-Abl parameters in peripheral blood and (2) alternative doses beyond these two are shown by the simulations in this work to be definitively suboptimal for achieving efficacy targets within well-tolerated exposure limits.

For non-pregnant patients, Johnson-Ansah e*t al.* demonstrated that continuously maintaining C_min_ >1000 ng/mL through TDM resulted in significantly higher major molecular response (MMR) rates at three years (81.1% for the TDM arm vs. 66.1% for the control arm) [38]. Additionally, adherence to imatinib is a well-documented factor influencing treatment outcomes, with patients who maintain adherence rates > 90% demonstrating improved responses even after 72 months further supporting the importance of continuously maintaining adequate exposure [42]. Even within 28 days, dose-dependent effects on white blood cells are observed [27].

Treatment interruptions, including drug holidays, may be an option for pregnant patients who have already achieved deep molecular responses. However, for patients who have not yet attained MMR or exhibit suboptimal response, maintaining adequate imatinib exposure is critical - particularly since switching to an alternative tyrosine kinase inhibitor (TKI), a common strategy in non-pregnant patients, is not a viable alternative during pregnancy. While the concerns regarding fetal exposure are recognized, it is also essential to consider that inadequate maternal disease control could lead to worse outcomes, including an increased risk of disease progression, which may necessitate more aggressive treatments post-pregnancy. Thus, balancing maternal treatment efficacy with fetal safety remains a key consideration. Therefore we advise, based on our simulations, that for all pregnant patients the initial starting dose should be 200 mg BID, who have not yet achieved MMR. While for patients with MMR, the registered dose in the label of 400 mg QD could be maintained throughout pregnancy. When TDM is available, dosing can be readily fine-tuned based on measured plasma concentrations. Continued lack of haematological response should be evaluated on a case-by-case basis, recognizing that (a) this model predicts that overall AUC_24SS_ following 300 mg BID dosing may be higher than that observed following 600 mg QD dosing (for a yet unexplained reason) and (b) the time course for achieving adequate response may extend beyond the pregnancy period – dose adjustment in pregnancy should only be practiced when there would be ≥ 28 days of the pregnant period remaining.

Long-term safety and pregnancy outcomes following a modified dosing scheme should be obtained by larger registry studies or even prospective clinical trials. In May 2019, the U.S. Food and Drug Administration (FDA) released draft guidance concerning post-approval pregnancy safety studies. This guidance offers detailed recommendations aimed at designing and conducting research to evaluate the effects of drugs and biological products on pregnancy outcomes. The guidance specifies how researchers should assess the outcomes of pregnancies in women who have been exposed to these substances. Furthermore, it provides a framework for incorporating findings from post-approval pregnancy safety studies or registries into the product labeling. This inclusion is crucial for ensuring that healthcare providers have access to up-to-date and comprehensive safety information. Such information is essential for advising patients who are pregnant or may become pregnant, thereby enhancing informed clinical decisions regarding the use of these drugs or biological products during pregnancy.

The work is not without limitations. Limited data was available to validate the pregnancy-PBPK model. Three concentration-time points were available from three pregnant women in the third trimester, showing mostly accurate predictions (i.e., no clear or systematic misspecification). No data was available for validation in the second trimester and so dosing simulations were not performed there. Future research should investigate fetal drug exposures by extending the pregnancy-PBPK model with fetal compartments in combination with maternal-to-fetal and fetal-to-maternal transfer ratios obtained from ex vivo placenta perfusion models [43].

Additional utility of the pregnancy PBPK model may lie in the simulation of imatinib drug-drug interactions in this population, as outlined by Leong e*t al*.^24^ Imatinib and its major metabolite act as inhibitors of CYP2C8, CYP2D6, and CYP3A4, and imatinib itself is victim to modulation of CYP3A4, CYP2C8, and P-gp.^28^ The files validated for simulation of drug-drug interactions by Loer et al. and then adapted for pregnancy are provided in the **Supplementary Material**.

## Conclusion

The developed pregnancy-PBPK model for imatinib well predicted the PK of imatinib in the third trimester of pregnancy and was used to simulate target attainment at the commonly used/standard dosage regimen (400 mg QD), as well as other dosing regimens that could possibly improve target attainment without increasing plasma AUC_0-24 h_. Simulations indicated that while dividing 400 mg QD into 200 mg BID could modestly improve PK target attainment for third trimester pregnant women (28.5%), a dose of 300 mg BID would be needed to match C_min_ target attainment for third trimester pregnant women (54.2%) and AUC_0-24 h_ with expectations for a non-pregnant population receiving 400 mg QD. However, there is minimal information to support the maternal-fetal safety of this increased total daily dose (600 mg total daily dose, and with AUC_24SS_ following 300 mg BID projected to be modestly higher than 600 mg QD). Intrauterine death at 7 months was reported for one mother-infant pair exposed to 600 mg QD and premature delivery with subsequent neonatal death was reported for two mother-infant pairs exposed to 800 mg QD.^7^ Further discussions on an updated imatinib dose in pregnant women may be warranted.

## Data Availability

No datasets were generated or analysed during the current study.

## Declarations

### Permission to reproduce materials from other sources

The authors give permission to reproduce materials from other sources.
